# Short- and Long-Term Outcomes of Neoadjuvant Chemotherapy in Operable Locally Advanced Colon Cancer: A Systematic Review

**DOI:** 10.7759/cureus.95339

**Published:** 2025-10-24

**Authors:** Adil Mohamed Ali Hassan Ahmed, Ammar Elnour, Fatima Aljenaid, Salma Bakri Hassan Mahmuod, Hager Elsir Sherfeldin Mohammed, Sami Mohammed Elhassan Elsafi Osman, Saba Hamad, Musab Mukhtar

**Affiliations:** 1 Medical Services, Catrion, Riyadh, SAU; 2 Gastroenterology, Barking, Havering and Redbridge University Hospitals NHS Trust, Romford, GBR; 3 General Practice, Princeway Health Centre, Frodsham, GBR; 4 Acute Medicine, Midland Metropolitan University Hospital, Smethwick, GBR; 5 Hepatology, King Abdulaziz Medical City - Riyadh, Riyadh, SAU; 6 School of Medicine, St. George's University, St. George's, GRD; 7 Gastroenterology, Lancashire Teaching Hospitals NHS Foundation Trust, Preston, GBR; 8 Internal Medicine, Sheikh Khalifa Specialty Hospital, Ras Al Khaimah, ARE

**Keywords:** colon cancer, locally advanced, neoadjuvant chemotherapy, overall survival, pathological complete response, systematic review

## Abstract

The management of operable locally advanced colon cancer has traditionally centered on upfront surgical resection. The role of neoadjuvant chemotherapy (NAC) in this setting remains a subject of investigation, with potential benefits including tumor downstaging and early treatment of micrometastases. This systematic review aims to synthesize the existing evidence on the short- and long-term outcomes of NAC for patients with operable locally advanced colon cancer. A systematic literature search was conducted across PubMed/MEDLINE, Embase, Scopus, and Web of Science up to October 2025, following Preferred Reporting Items for Systematic Reviews and Meta-Analyses (PRISMA) guidelines. Studies reporting on pathological response, surgical outcomes, recurrence, disease-free survival (DFS), or overall survival (OS) in patients receiving NAC for locally advanced colon cancer were included. The risk of bias was assessed using the Cochrane RoB 2 tool for randomized trials and the ROBINS-I tool for non-randomized studies. A qualitative synthesis was performed due to heterogeneity among the included studies. Twelve studies were included. Pathological complete response rates varied, reaching up to 16% with FOLFOX-based regimens in colon cancer. NAC was associated with high R0 resection rates and acceptable postoperative morbidity. A key finding was the stage-dependent survival benefit, with a significant OS improvement specifically in T4 disease but not in T3 disease. The addition of targeted therapy based on biomarker status (e.g., panitumumab in KRAS-wildtype tumors) demonstrated significant improvements in DFS and OS. Evidence from rectal cancer studies suggested that NAC could achieve outcomes comparable to neoadjuvant chemoradiotherapy. NAC is a feasible and effective strategy for operable locally advanced colon cancer, demonstrating significant pathological responses and promising survival outcomes, particularly in T4 tumors and with biomarker-directed therapy. Its efficacy is highly dependent on careful patient selection based on disease stage and molecular characteristics. Future research should focus on randomized trials in high-risk populations and the integration of personalized treatment approaches.

## Introduction and background

Colorectal cancer is the third most commonly diagnosed malignancy worldwide and a leading cause of cancer-related mortality [1]. Among its subtypes, colon cancer accounts for the majority of cases, with a substantial proportion of patients presenting at a locally advanced stage. Traditionally, the standard treatment for operable locally advanced colon cancer has been upfront surgical resection, followed by adjuvant chemotherapy (NAC) when indicated [2]. While this approach has improved survival, challenges persist, including high rates of postoperative morbidity, incomplete delivery of adjuvant therapy, and the continued risk of local recurrence and distant metastases. These limitations raise the question of whether the current approach has reached the horizon of its maximal efficacy [3].

In recent years, NAC, particularly FOLFOX, has gained attention as a potential strategy to optimize outcomes in locally advanced colon cancer [4]. Administered before surgery, NAC may facilitate tumor downstaging, increase the likelihood of achieving R0 resection, and target micrometastatic disease early in the treatment course [5]. Preoperative systemic therapy may also be better tolerated, improving treatment adherence. Beyond these benefits, NAC could modulate the immune response against the primary tumor, potentially enhancing anti-tumor immunity and influencing long-term outcomes. Insights from rectal cancer and other solid tumors further support exploring neoadjuvant strategies in colon cancer management [6].

With the expanding therapeutic landscape, the concept of neoadjuvant treatment now extends beyond conventional cytotoxic regimens. Investigations into FOLFIRI and less toxic fluoropyrimidine-based combinations have suggested comparable efficacy in selected patients with improved tolerability. Moreover, the integration of targeted agents and immunotherapy is reshaping the paradigm of systemic therapy in colorectal malignancies. Biomarker-driven approaches, particularly those guided by microsatellite instability, mismatch repair status, and RAS or BRAF mutations, have demonstrated promising activity in advanced disease and may hold future potential in the neoadjuvant setting for operable tumors [7].

Despite these advances, the role of NAC in operable locally advanced colon cancer remains debated. Concerns include treatment-related toxicity, the risk of disease progression during chemotherapy, and possible delays in curative surgery. Furthermore, it remains uncertain which outcomes should be prioritized: while overall survival (OS) is conventionally considered the most critical endpoint, secondary outcomes such as disease-free survival (DFS), pathological response, surgical feasibility, and perioperative morbidity are also highly relevant for assessing the full therapeutic impact of NAC. Given that most existing evidence focuses predominantly on FOLFOX-based regimens, it is important to clarify this from the outset.

Given the evolving treatment landscape and growing clinical interest, a comprehensive appraisal of current evidence is warranted. This systematic review aims to evaluate both short- and long-term outcomes of neoadjuvant FOLFOX in operable locally advanced colon cancer, focusing on pathological response, surgical feasibility, perioperative morbidity and mortality, recurrence patterns, DFS, and OS. By critically examining the available data, this review seeks to clarify the therapeutic value of NAC in this patient population and provide insights that may inform future clinical practice and research.

## Review

Methodology

Eligibility Criteria

This systematic review was conducted following the Preferred Reporting Items for Systematic Reviews and Meta-Analyses (PRISMA) guidelines [8]. We included original studies published until October 2025 that investigated the impact of NAC on short- and long-term outcomes in patients with operable locally advanced colon cancer. Eligible studies were randomized controlled trials (RCTs), prospective cohort studies, and retrospective observational studies that reported at least one of the following outcomes: pathological response, R0 resection rate, postoperative morbidity and mortality, recurrence, DFS, or OS. Only studies published in English and involving human subjects were considered. Case reports, editorials, conference abstracts, reviews, and studies focusing on rectal cancer or metastatic colon cancer were excluded.

Information Sources and Search Strategy

A comprehensive literature search was conducted in four major databases: PubMed/MEDLINE, Embase (Elsevier), Scopus, and Web of Science. The search strategy combined keywords and Medical Subject Headings (MeSH) related to “colon cancer,” “neoadjuvant chemotherapy,” and “locally advanced disease.” Boolean operators (AND, OR) were used to refine the searches. To maximize study retrieval, we also performed citation searching of included studies and relevant reviews to identify additional eligible articles. The final search was completed in October 2025.

Selection Process

All retrieved records were imported into EndNote X9 (Clarivate Analytics, Philadelphia, USA) for duplicate removal. Two independent reviewers screened titles and abstracts for relevance. Full texts of potentially eligible studies were then reviewed against the inclusion and exclusion criteria. Discrepancies between reviewers were resolved by consensus or consultation with a third reviewer. A PRISMA flow diagram was used to document the study selection process.

Data Collection Process

Data extraction was performed independently by two reviewers using a standardized data extraction form. Extracted information included study characteristics (first author, year, country, study design, sample size), patient population details, NAC regimens, comparator groups (if any), follow-up duration, and reported short- and long-term outcomes. Any disagreements were resolved by discussion.

Data Items

The outcomes of interest were grouped into two main categories: (1) short-term outcomes, including pathological complete response (pCR), tumor downstaging, R0 resection rates, postoperative morbidity, and mortality, and (2) long-term outcomes, including local recurrence rates, DFS, and OS.

Study Risk of Bias Assessment

The risk of bias was assessed at the study level. For RCTs, the Revised Cochrane Risk of Bias tool (RoB 2) was used [9], while non-randomized studies were evaluated using the Risk of Bias in Non-randomized Studies of Interventions (ROBINS-I) tool [10]. Assessments were performed independently by two reviewers, and disagreements were resolved through consensus.

Synthesis of Results

Given the heterogeneity across studies in terms of patient selection, chemotherapy regimens, outcome definitions, and follow-up duration, a meta-analysis was not performed. Instead, a qualitative synthesis was undertaken to summarize the evidence narratively. This decision was made to avoid misleading pooled estimates that could arise from combining highly diverse data and to preserve the clinical context of each study.

Results

Study Selection Process

**Figure 1:**
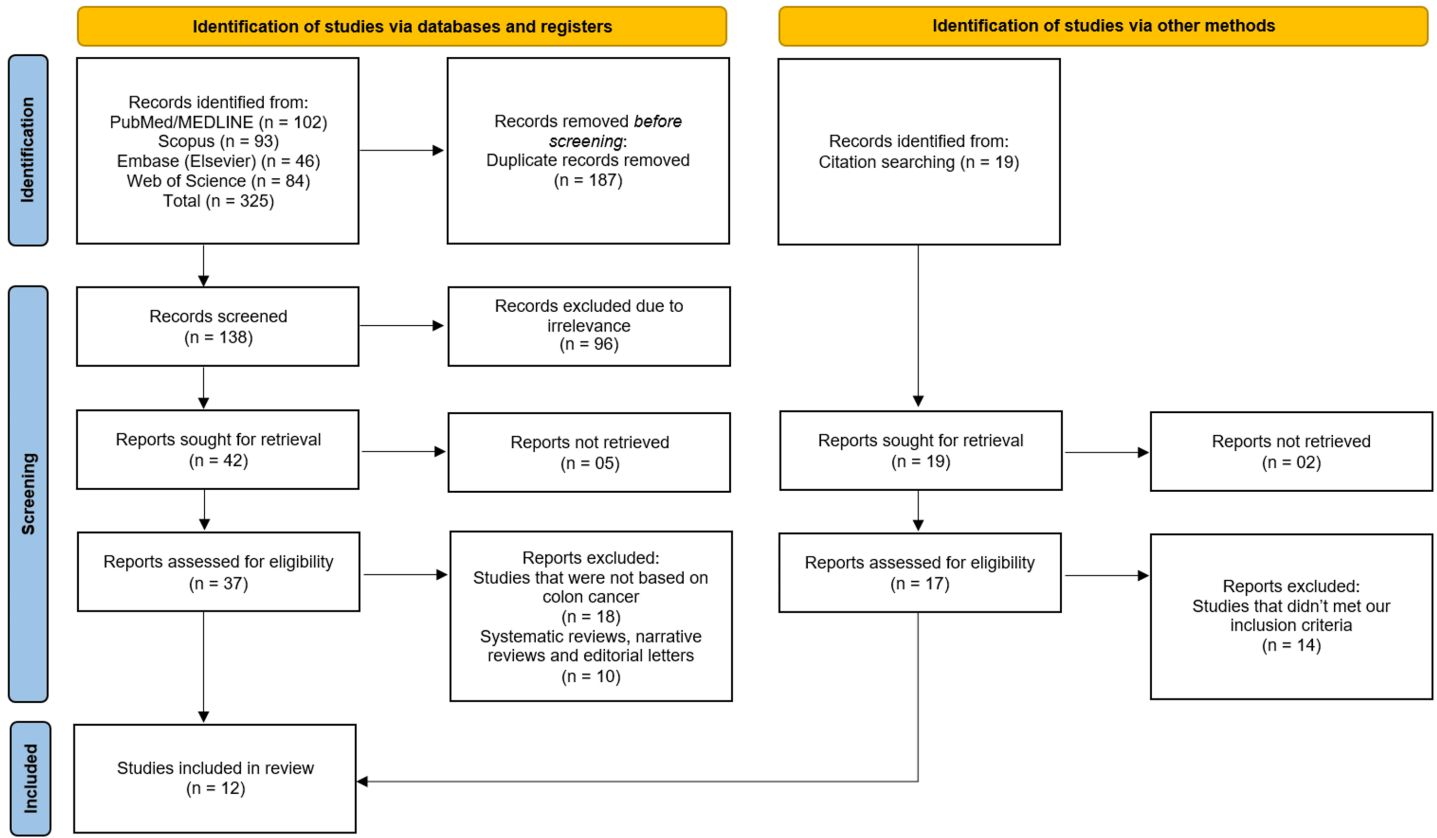
Schematic of the study selection process according to the PRISMA guidelines PRISMA: Preferred Reporting Items for Systematic Reviews and Meta-Analyses

The systematic search across four electronic databases (PubMed/MEDLINE, Scopus, Embase, and Web of Science) initially identified 325 records. After the removal of 187 duplicate records, 138 unique studies were screened based on their titles and abstracts. This led to the exclusion of 96 records due to irrelevance. A total of 42 full-text articles were sought for retrieval, of which 37 were successfully accessed. For the five articles that could not be retrieved due to paywall restrictions, we contacted the corresponding authors via email but did not receive any response. These 37 full-text articles were assessed for eligibility against the pre-defined inclusion criteria. Subsequently, 28 of these articles were excluded, with the primary reasons being a focus on cancer types other than colon cancer (n=18) and the publication type being systematic reviews, narrative reviews, or editorial letters (n=10). An additional 19 records were identified through citation searching, of which 17 were assessed at the full-text stage after two reports could not be retrieved. We also contacted the corresponding authors of these two studies, but no reply was received. From this secondary pool, 14 studies were excluded for not meeting the inclusion criteria. Ultimately, this rigorous selection process resulted in 12 studies [11-22] being included in the qualitative synthesis of this systematic review (Figure 1).

Study Characteristics

A total of 12 studies [11-22] were included in this systematic review, comprising a mix of RCTs, phase II trials, and retrospective cohort analyses, published between 2018 and 2025. The characteristics of these studies are summarized in Table 1. The sample sizes varied considerably, ranging from 26 participants in a small comparative cohort [20] to a large nationwide analysis of 60,557 patients [12]. Geographically, the studies were conducted in East Asia (China [11,15,16,18,19], Taiwan [17], Japan [20,21]), North America (United States [12,14,22], and Europe (United Kingdom [13]).

**Table 1:** Characteristics of Included Studies OS: Overall survival; PFS: progression-free survival; RFS: recurrence-free survival; NAC: neoadjuvant chemotherapy; NACRT: neoadjuvant chemoradiotherapy

First Author (Year)	Country / Region	Study Design	Sample Size (n)	Patient Population	Neoadjuvant Chemotherapy Regimen	Comparator (if any)	Follow-Up Duration
Zhou et al., [11] (2025)	China (West China Hospital, Sichuan University)	Single-arm, open-label, phase II trial	72	Patients with hr-LACC (T4a/b and/or N2/fused lymph nodes), no distant metastases; median age 53 (18–74)	FOLFOXIRI (4 cycles)	None (single-arm)	2 years
Pena et al., [12] (2025)	United States (National Cancer Database, 2010–2020)	Nationwide retrospective analysis	60,557 (NAC: 2,313; Upfront Surgery: 58,245)	Patients with clinical stage T3 and T4 colon adenocarcinoma	Neoadjuvant chemotherapy	Upfront surgery followed by standard adjuvant chemotherapy	Data derived from NCDB 2010–2020
Seligmann et al., [13] (2025)	United Kingdom / FOxTROT Trial (multicenter)	Randomised Phase II Trial (embedded within FOxTROT)	269 KRAS-wt patients (232 with extended RAS/BRAF data; 169 RAS/BRAF-wt included in efficacy analysis)	Operable, CT-predicted stage T3–4, N0–2, M0 colon adenocarcinoma; RAS/BRAF-wt subgroup assessed	Neoadjuvant FOLFOX ± Panitumumab (biomarker hyperselected patients also analyzed by EREG/AREG expression)	FOLFOX alone	Median follow-up: 42 months
Schrag et al., [14] (2023)	United States (multicenter, North America)	Randomized controlled trial (noninferiority, multicenter, unblinded)	1128 (585 FOLFOX; 543 chemoradiotherapy)	Adults with locally advanced rectal cancer (cT2N+, cT3N0, cT3N+) eligible for sphincter-sparing surgery	FOLFOX (fluorouracil, leucovorin, oxaliplatin) ± chemoradiotherapy if <20% tumor shrinkage or toxicity	Preoperative chemoradiotherapy (fluoropyrimidine-based)	Median 58 months
Mei et al., [15] (2023)	China	Randomized controlled trial	589 (nCT: 300; nCRT: 289)	Patients with locally advanced rectal cancer within 12 cm from anal verge, uninvolved mesorectal fascia	nCT: 4 cycles CAPOX (Oxaliplatin 130 mg/m² IV day 1 + Capecitabine 1000 mg/m² twice daily for 14 days, repeated every 3 weeks)	nCRT: Capecitabine 825 mg/m² twice daily + radiation therapy (50 Gy/25 fractions, 5 days/week)	NR
Han et al., [16] (2023)	China	Retrospective	155	Patients with LARC	Median 4 cycles of nCT	nCRT: 50 Gy/25 Fx + concurrent capecitabine (median 2 cycles of nCT)	30 months
Yin et al., [17] (2023)	Taiwan	Retrospective cohort	63	Patients with LARC and synchronous metastasis	Systemic chemotherapy + targeted therapy ± concurrent radiotherapy	RT-CT group (chemo + targeted therapy + radiotherapy) vs CT group (chemo + targeted therapy only)	PFS reported up to 22.5 months
Zhao et al., [18] (2022)	China	Retrospective cohort	184 (92 per group)	Patients with LARC	pNCT: 6–8 cycles CapeOX/FOLFOX	TNT: 4 cycles induction CapeOX/FOLFOX + CRT	Median 35 months
Deng et al., [19] (2019)	China	Multicenter, open-label, phase III RCT	495	Adults 18–75 years with stage II/III rectal cancer	1) Infusional fluorouracil + leucovorin + radiotherapy; 2) mFOLFOX6 + radiotherapy; 3) mFOLFOX6 alone	Fluorouracil + radiotherapy (arm 1)	Median 45.2 months
Sato et al., [20] (2019)	Japan	Comparative cohort	26 (16 NAC, 10 NACRT)	Patients with lower rectal cancer undergoing preoperative therapy	NAC	NACRT	NR
Okuyama et al., [21] (2018)	Japan	Retrospective cohort study	55 (27 NAC, 28 NACRT)	Patients with cT3/4 and N+ locally advanced rectal cancer	Oxaliplatin-based NAC (2–9 cycles)	NACRT (45 Gy in 25 fractions + 5-FU-based oral chemotherapy)	Three-year RFS; four-year OS
Sada et al., [22] (2018)	USA	Retrospective cohort	12,024	Patients aged 18–80 y with clinical stage II–III rectal adenocarcinoma	Neoadjuvant chemotherapy alone	Neoadjuvant radiation	Five-year OS reported

The patient populations primarily consisted of individuals with locally advanced rectal cancer (LARC) [14-22], with three studies focusing specifically on colon cancer [11-13]. The NAC regimens were predominantly based on fluoropyrimidine and oxaliplatin, such as FOLFOX, CAPOX, or FOLFOXIRI. Common comparators included nCRT [14-18,20,21] and upfront surgery [12]. The follow-up duration also varied, with median follow-up times ranging from 30 months [16] to 58 months [14] in studies that reported it.

Pathological and Surgical Outcomes

pCR and tumor downstaging were key short-term endpoints reported across several studies. The pCR rates for NAC regimens varied, with reports of 7.8% with FOLFOXIRI in high-risk colon cancer [11], 11.0% with CAPOX in rectal cancer [15], and 16% in the FOLFOX-alone arm for colon cancer [13]. The addition of panitumumab to FOLFOX in KRAS-wildtype colon cancer patients showed a non-significant trend towards improved tumor regression (16% vs 22% achieving regression grades 1-3) [13]. In rectal cancer, one study comparing prolonged NAC to total neoadjuvant therapy (TNT) reported higher pCR rates with the TNT approach (25% vs 16.3%) [18]. Furthermore, a study by Sada et al. highlighted the prognostic importance of treatment response, showing that patients with a complete pathological response had superior OS [22].

In terms of surgical outcomes, a very high R0 resection rate of 100% was reported in a phase II trial of FOLFOXIRI for colon cancer [11]. Postoperative morbidity was generally low; for instance, Zhou et al. reported a 4.7% rate of prolonged hospital stay due to complications and no 30- or 90-day mortality [11]. The comparative safety of NAC versus nCRT was a focus in several studies, with one reporting no significant difference in postoperative morbidity between the two approaches [16].

Survival and Recurrence Outcomes

The data on survival outcomes revealed nuanced findings. In the large retrospective analysis by Pena et al., the impact of NAC on OS in colon cancer was stage-dependent. While NAC was associated with worse OS in the overall T3/T4 cohort (HR 1.16), subgroup analysis revealed a significant survival benefit specifically in T4 disease (HR 0.85) and no benefit in T3 disease [12]. For rectal cancer, the large randomized trial by Schrag et al. found that preoperative FOLFOX was non-inferior to nCRT, with no significant difference in five-year DFS (DFS: 80.8% vs 78.6%) or OS between the groups [14]. Similarly, the FOWARC trial found comparable three-year DFS and OS across arms of fluorouracil+RT, mFOLFOX6+RT, and mFOLFOX6 alone [19].

The efficacy of adding targeted therapy was evaluated in the FOxTROT trial, where the addition of panitumumab to FOLFOX in selected colon cancer patients led to a significant improvement in both DFS and OS [13]. Recurrence patterns were also assessed. Local recurrence rates at two years were 17.2% in a colon cancer trial [11] and 16.7% in a rectal cancer study [16]. Yin et al. reported that the addition of concurrent radiotherapy to systemic therapy in LARC patients with synchronous metastases prolonged local recurrence-free survival and progression-free survival (PFS 22.5 months vs 13.3 months) [17]. Okuyama et al. reported favorable long-term outcomes with oxaliplatin-based NAC, with a three-year recurrence-free survival of 85.2% and a four-year OS of 96.3% [21]. The short- and long-term outcomes of all included studies are detailed in Table 2.

**Table 2:** Short- and Long-Term Outcomes of Neoadjuvant Chemotherapy

First Author (Year)	Pathological Response (pCR/Tumor Downstaging %)	R0 Resection Rate (%)	Postoperative Morbidity (%)	Postoperative Mortality (%)	Local Recurrence Rate (%)	Disease-Free Survival (DFS) (%)	Overall Survival (OS) (%)	Median Follow-Up (Months/Years)
Zhou et al., [11] (2025)	pCR: 7.8% (5/64); TRG 0–2: 87.5%; pT0–2: 21.9%; pN0: 68.8%	100% (64/64)	4.7% (prolonged hospital stay due to complications)	0% (no 30- or 90-day mortality)	17.2% (two-year recurrence)	NR	NR	Median FU: 2 years
Pena et al., [12] (2025)	NR	NR	NR	NR	NR	NR	OS: Worse in NAC group overall (HR 1.16, 95% CI 1.07–1.26); No benefit in T3 (HR 1.13, 95% CI 0.99–1.28); Significant benefit in T4 (HR 0.85, 95% CI 0.77–0.95)	NR
Seligmann et al., [13] (2025)	Tumor regression grade 1–3: 16% (FOLFOX+Pani) vs 22% (FOLFOX) (NS)	NR	NR but ↑ grade 3 diarrhea 8% vs 3%, rash 22% vs 2%	NR	Recurrence: 12% (FOLFOX+Pani) vs 21% (FOLFOX)	Significant improvement with panitumumab	Significant improvement with panitumumab	42 months
Schrag et al., [14] (2023)	NR	NR	NR	NR	Similar between groups (HR 1.18; 95% CI 0.44–3.16)	5-yr DFS: 80.8% (FOLFOX) vs 78.6% (CRT)	Similar between groups (HR 1.04; 95% CI 0.74–1.44)	Median 58 months
Mei et al., [15] (2023)	nCT (CAPOX) vs nCRT (Capecitabine + RT)	pCR 11.0% / 40.8% vs 13.8% / 45.6%	NR	NR	NR	NR	NR	NR
Han et al., [16] (2023)	5.6% pCR	NR	No significant difference	NR	16.7%	No significant difference	No significant difference	30 months
Yin et al., [17] (2023)	NR	RT-CT: 71.4CT: 42.9	Similar between groups; diarrhea higher in RT-CT (59.5% vs 23.8%)	NR	RT-CT: longer local recurrence-free survival	RT-CT: PFS 22.5 moCT: PFS 13.3 mo	No significant difference between groups	NR
Zhao et al., [18] (2022)	TNT: pCR 25%, Tumor regression 73.9%pNCT: pCR 16.3%, Tumor regression 59.8%	NR	NR	NR	NR	TNT & pNCT: similar three-year RFS	TNT & pNCT: similar three-year OS	35 months
Deng et al., [19] (2019)	Fluorouracil + RT / mFOLFOX6 + RT / mFOLFOX6	NR	NR	NR	NR	8.0/7.0/8.3	72.9/77.2/73.5	91.3/89.1/90.7
Sato et al., [20] (2019)	pCR (Primary): 12.5%T Downstaging: 87.5%pT Downstaging: 25%pN Conversion: 80%	NR	NR	NR	NR	NR	NR	NR
Okuyama et al., [21] (2018)	NR	NR	NR	NR	7.4	Three-year RFS 85.2% (all NAC); 94.4% (excluding adjuvant chemo)	Four-year OS 96.3%	Median follow-up not reported; three-year and four-year outcomes reported
Sada et al., [22] (2018)	Complete: 12%Intermediate: 30%	NR	NR	NR	NR	NR	Complete: 90.2%; Intermediate: 82.0%; No response: 70.5%	NR

Risk of Bias Assessment

The methodological quality of the included studies was appraised using the Cochrane RoB 2 tool for randomized trials and the ROBINS-I tool for non-randomized studies. Among the four randomized controlled trials [13-15,19], three were judged to have a low risk of bias overall [14,15,19], while the trial by Seligmann et al. [13] was assessed as having some concerns, primarily due to potential deviations from the intended interventions. For the eight non-randomized studies [11,12,16-18,20-22], the risk of bias was predominantly low, with the exception of the study by Han et al. [16], which was rated as having a serious risk of bias due to confounding. This indicates that the body of evidence from the RCTs is robust, and the majority of the non-randomized studies also demonstrate a low susceptibility to bias (Tables 3, 4).

**Table 3:** Risk of Bias Assessment for Randomized Controlled Trials (Using the RoB 2 Tool) RoB: Risk of bias

First Author, Year	Randomization Process	Deviations from Intended Interventions	Missing Outcome Data	Measurement of the Outcome	Selection of the Reported Result	Overall Bias
Seligmann et al., [13] (2025)	Low	Some Concerns	Low	Low	Low	Some Concerns
Schrag et al., [14] (2023)	Low	Low	Low	Low	Low	Low
Mei et al., [15] (2023)	Low	Low	Low	Low	Low	Low
Deng et al., [19] (2019)	Low	Low	Low	Low	Low	Low

**Table 4:** Risk of Bias Assessment for Non-Randomized Studies (Using the ROBINS-I Tool) ROBINS-I:  Risk of Bias in Non-randomized Studies of Interventions

First Author, Year	Confounding	Selection of Participants	Classification of Interventions	Deviations from Intended Interventions	Missing Data	Measurement of Outcomes	Selection of the Reported Result	Overall Bias
Zhou et al., [11] (2025)	Low	Low	Low	Low	Low	Low	Low	Low
Pena et al., [12] (2025)	Low	Low	Low	Low	Low	Low	Low	Low
Han et al., [16] (2023)	Serious	Low	Low	Low	Low	Low	Low	Serious
Yin et al., [17] (2023)	Low	Low	Low	Low	Low	Low	Low	Low
Zhao et al., [18] (2022)	Low	Low	Low	Low	Low	Low	Low	Low
Sato et al., [20] (2019)	Low	Low	Low	Low	Low	Low	Low	Low
Okuyama et al., [21] (2018)	Low	Low	Low	Low	Low	Low	Low	Low
Sada et al., [22] (2018)	Low	Low	Low	Low	Low	Low	Low	Low

Discussion

This systematic review synthesizes evidence from 12 studies [11-22] to evaluate the short- and long-term outcomes of NAC in operable locally advanced colon cancer. The findings reveal a complex and evolving landscape, indicating that while NAC is a feasible and promising strategy, its benefits are not uniform across all patient subgroups and are highly dependent on disease stage, tumor biology, and the specific therapeutic regimen employed. The collective evidence suggests that NAC can induce significant pathological responses, facilitate successful surgical resection, and potentially alter the natural history of the disease, particularly in the most advanced cases. However, the translation of these short-term benefits into unequivocal long-term survival advantages remains nuanced and requires careful patient selection.

The pathological response, particularly the rate of pCR, serves as a critical early indicator of treatment efficacy. The observed pCR rates in this review, ranging from 7.8% with FOLFOXIRI [11] to 16% with FOLFOX [13] in colon cancer-specific studies, are consistent with earlier investigations into intensive chemotherapy regimens. For instance, the landmark FOXTROT pilot study, a precursor to the work by Seligmann et al. [13], initially demonstrated the feasibility of NAC in colon cancer and reported a pCR rate of approximately 4%, with modern iterations showing improved rates with optimized regimens. The higher pCR rate of 25% associated with TNT in rectal cancer, as reported by Zhao et al. [18], underscores the potential of intensifying preoperative treatment, a concept that is now being actively explored in colon cancer. The prognostic significance of achieving a good pathological response is powerfully highlighted by the work of Sada et al. [22], which demonstrated a clear gradient in OS favoring patients with complete response. This echoes findings from studies in rectal cancer, such as those by Maas et al. [23], which established pCR as a strong surrogate for superior long-term outcomes, suggesting that response-adapted strategies could be a future direction for colon cancer management.

A pivotal finding of this review is the stark stage-dependent survival benefit of NAC, as elucidated by the large-scale analysis of Pena et al. [12]. The observation that NAC was associated with a significant survival benefit in T4 disease (HR 0.85) but not in T3 disease, and was even detrimental in the overall cohort, is of paramount clinical importance. This can be interpreted through the lens of tumor biology and micrometastatic burden. T4 tumors, by virtue of their penetration through the colonic wall, are at a substantially higher risk of harboring occult metastatic disease. In this context, the early introduction of systemic chemotherapy with NAC may be more effective at eradicating micrometastases than adjuvant chemotherapy given after surgical recovery, a period during which resistant clones may have already been seeded. This hypothesis is supported by the long-standing principles of the "Norton-Simon" model, which posits that chemotherapy is most effective against minimal disease burden [24]. Conversely, for T3 disease, the risks of chemotherapy-related toxicity and potential for delaying curative surgery may outweigh the benefits in a population with a lower baseline risk of systemic recurrence. This finding necessitates a paradigm shift away from a one-size-fits-all approach and towards a more precise, stage-specific utilization of NAC.

The surgical implications of NAC are another crucial aspect. The 100% R0 resection rate reported by Zhou et al. [11] and the generally low postoperative morbidity are highly encouraging. The primary goal of NAC in solid tumors is often to downstage the tumor, facilitating a more complete and less morbid resection. In the context of colon cancer, where achieving a clear radial margin is critical, NAC may be particularly beneficial for tumors with suspected involvement of surrounding structures or those deemed borderline resectable. This aligns with the rationale behind using NAC in other gastrointestinal malignancies, such as gastric cancer, where studies like the MAGIC trial demonstrated increased R0 resection rates with perioperative chemotherapy [25]. The low mortality rates reported across the studies, notably the 0% 30- or 90-day mortality in the trial by Zhou et al. [11], affirm the safety of this approach in well-selected patients within experienced centers, allaying one of the primary concerns regarding the preoperative delay of surgery.

When comparing NAC to established neoadjuvant strategies, the data from rectal cancer studies included in this review provide invaluable insights. The randomized trial by Schrag et al. [14] represents a landmark, demonstrating the non-inferiority of NAC with FOLFOX compared to nCRT in terms of DFS and OS. This challenges the long-standing dominance of nCRT as the standard of care for LARC and suggests that for a subset of patients, systemic chemotherapy alone may be sufficient to control both local and distant disease. This is further reinforced by the FOWARC trial [19], which showed comparable survival outcomes between mFOLFOX6-based regimens and fluorouracil-based chemoradiation. These findings have profound implications for colon cancer, where radiotherapy plays a minimal role. They validate the concept that potent systemic therapy can effectively address the primary tumor without the added toxicity of radiation, supporting the investigation of NAC as a primary preoperative modality. However, the superior local control suggested by the addition of radiotherapy in the study by Yin et al. [17] indicates that for specific high-risk scenarios, such as cases with threatened margins, a multimodal approach including radiation may still hold value, mirroring the ongoing debate in rectal cancer management.

Perhaps the most forward-looking finding is the demonstration of personalized therapy through biomarker integration, as seen in the FOxTROT trial analysis by Seligmann et al. [13]. The significant improvement in DFS and OS with the addition of panitumumab to FOLFOX in carefully selected KRAS-wildtype patients, and further refined by EREG/AREG expression, represents the pinnacle of precision oncology in the neoadjuvant setting. This aligns with the established role of anti-EGFR therapy in metastatic colorectal cancer but pushes the frontier into earlier disease stages. It suggests that the future of NAC lies not merely in chemotherapy selection, but in leveraging tumor genetics to identify patients most likely to benefit from targeted agents. This approach is reminiscent of the successful integration of trastuzumab in neoadjuvant breast cancer regimens, where biomarker selection led to dramatic improvements in pCR and survival. The non-significant trend in pathological response in the Seligmann study, contrasted with the clear survival benefit, also underscores the limitation of pCR as a sole surrogate endpoint and highlights the importance of targeting micrometastatic disease, which is not fully captured by pathological grading of the primary tumor.

Despite these promising findings, it is crucial to contextualize them within the broader literature. Our results both confirm and challenge previous understandings. The stage-specific benefit for T4 tumors [12] corroborates the hypothesis generated by earlier smaller studies and meta-analyses, such as the one by Hu et al. [26], which suggested a more pronounced benefit for higher-risk disease. However, the lack of benefit in T3 disease contradicts more optimistic projections from some single-arm phase II trials, emphasizing the necessity of robust, large-scale comparative data. The pCR rates we report, while modest, are generally higher than those seen in the initial pilot studies of NAC for colon cancer, likely reflecting the use of more modern and intensive chemotherapy backbones like FOLFOXIRI and CAPOX. Furthermore, the successful application of NAC in rectal cancer as a viable alternative to nCRT [14,19] provides a strong, parallel evidence base that supports its biological rationale in colon cancer, reinforcing the concept of "total neoadjuvant" systemic therapy. The safety profile observed across studies is also consistent with the known toxicity spectra of these regimens and aligns with safety data from their use in the metastatic and adjuvant settings, as documented in trials like MOSAIC.

This systematic review is subject to several limitations. Firstly, the included studies are heterogeneous in design, patient population (with a majority focusing on rectal cancer), and chemotherapeutic regimens, which precluded a formal meta-analysis and complicates direct comparisons. Secondly, as reflected in the risk of bias assessment, the evidence is heavily weighted towards non-randomized studies, which are inherently susceptible to confounding by indication; for example, patients selected for NAC may have had more favorable performance status or different tumor characteristics than those undergoing upfront surgery. The single serious risk of bias rating for the study by Han et al. [16] due to confounding is a particular concern within this body of evidence. While the RCTs were generally of high quality, the "some concerns" rating for the trial by Seligmann et al. [13] related to deviations from the protocol highlights the practical challenges in conducting neoadjuvant trials. Finally, the relatively short follow-up in some studies may not have captured late recurrences or long-term toxicities, potentially overestimating the durability of benefit.

## Conclusions

NAC is a viable and increasingly important strategy in the management of operable locally advanced colon cancer, particularly for patients with T4 disease. It demonstrates the capacity of NAC to induce significant tumor regression, facilitate complete surgical resection, and, when guided by robust biomarkers, improve survival outcomes. The critical lesson is that the efficacy of NAC is not universal but is contingent upon careful patient stratification based on the clinical stage and molecular characteristics. The success of NAC in rectal cancer provides a compelling template for its further development in colon cancer. Future research should focus on large, randomized trials in well-defined high-risk colon cancer populations, the continued integration of biomarker-driven therapy, and the exploration of TNT strategies to maximize both local and systemic disease control before surgery. Ultimately, the move towards a personalized, neoadjuvant approach promises to improve outcomes for patients with this challenging disease.

## References

[1] Xi Y, Xu P (2021). Global colorectal cancer burden in 2020 and projections to 2040. Transl Oncol.

[2] Ahmed M (2020). Colon cancer: a clinician’s perspective in 2019. Gastroenterology Res.

[3] Constantin VD, Silaghi A, Epistatu D, Dumitriu AS, Paunica S, Bălan DG, Socea B (2023). Diagnosis and management of colon cancer patients presenting in advanced stages of complications. J Mind Med Sci.

[4] Arredondo J, Pastor E, Simó V (2020). Neoadjuvant chemotherapy in locally advanced colon cancer: a systematic review. Tech Coloproctol.

[5] Liang Z, Li Z, Yang Q (2022). The role of neoadjuvant chemotherapy in patients with locally advanced colon cancer: a systematic review and meta-analysis. Front Oncol.

[6] Keller DS, Berho M, Perez RO, Wexner SD, Chand M (2020). The multidisciplinary management of rectal cancer. Nat Rev Gastroenterol Hepatol.

[7] Gosavi R, Chia C, Michael M, Heriot AG, Warrier SK, Kong JC (2021). Neoadjuvant chemotherapy in locally advanced colon cancer: a systematic review and meta-analysis. Int J Colorectal Dis.

[8] Page MJ, McKenzie JE, Bossuyt PM (2021). The PRISMA 2020 statement: an updated guideline for reporting systematic reviews. BMJ.

[9] Sterne JA, Savović J, Page MJ (2019). RoB 2: a revised tool for assessing risk of bias in randomised trials. BMJ.

[10] Sterne JA, Hernán MA, Reeves BC (2016). ROBINS-I: a tool for assessing risk of bias in non-randomised studies of interventions. BMJ.

[11] Zhou Y, Meng W, Qiu M (2025). Neoadjuvant chemotherapy with FOLFOXIRI for high-risk relapsed locally advanced colon cancer: a single-arm phase II trial. Am Soc Clin Oncol.

[12] Pena P, Sousa A, AlMasad Q, Kwon S, Williams R (2025). Neoadjuvant therapy in T3 and T4 colon cancer: is there a promising future?. Am Soc Clin Oncol.

[13] Seligmann JF, Morton D, Elliott F (2025). Neo-adjuvant FOLFOX with and without panitumumab for patients with KRAS-wt locally advanced colon cancer: results following an extended biomarker panel on the FOxTROT trial embedded phase II population. Ann Oncol.

[14] Schrag D, Shi Q, Weiser MR (2023). Preoperative treatment of locally advanced rectal cancer. N Engl J Med.

[15] Mei WJ, Wang XZ, Li YF (2023). Neoadjuvant chemotherapy with CAPOX versus chemoradiation for locally advanced rectal cancer with uninvolved mesorectal fascia (CONVERT): initial results of a phase III trial. Ann Surg.

[16] Han YM, Qi WX, Wang SB, Cao WG, Chen JY, Cai G (2023). Identification of patients with locally advanced rectal cancer eligible for neoadjuvant chemotherapy alone: results of a retrospective study. Cancer Med.

[17] Yin TC, Chen PJ, Yeh YS (2023). Efficacy of concurrent radiotherapy in patients with locally advanced rectal cancer and synchronous metastasis receiving systemic therapy. Front Oncol.

[18] Zhao X, Han P, Zhang L (2022). Prolonged neoadjuvant chemotherapy without radiation versus total neoadjuvant therapy for locally advanced rectal cancer: a propensity score matched study. Front Oncol.

[19] Deng Y, Chi P, Lan P (2019). Neoadjuvant modified FOLFOX6 with or without radiation versus fluorouracil plus radiation for locally advanced rectal cancer: final results of the Chinese FOWARC trial. J Clin Oncol.

[20] Sato K, Miura T, Morohashi S, Sakamoto Y, Morohashi H, Yoshida T, Hakamada K (2019). Comparable regional therapeutic effects between neoadjuvant chemotherapy and neoadjuvant chemoradiotherapy for locally advanced lower rectal cancer in terms of histopathological analysis. Mol Clin Oncol.

[21] Okuyama T, Sameshima S, Takeshita E (2018). Therapeutic effects of oxaliplatin-based neoadjuvant chemotherapy and chemoradiotherapy in patients with locally advanced rectal cancer: a single-center, retrospective cohort study. World J Surg Oncol.

[22] Sada YH, Tran Cao HS, Chang GJ, Artinyan A, Musher BL, Smaglo BG, Massarweh NN (2018). Prognostic value of neoadjuvant treatment response in locally advanced rectal cancer. J Surg Res.

[23] Maas M, Nelemans PJ, Valentini V (2010). Long-term outcome in patients with a pathological complete response after chemoradiation for rectal cancer: a pooled analysis of individual patient data. Lancet Oncol.

[24] Norton L, Simon R (1986). The Norton-Simon hypothesis revisited. Cancer Treat Rep.

[25] Cunningham D, Allum WH, Stenning SP (2006). Perioperative chemotherapy versus surgery alone for resectable gastroesophageal cancer. N Engl J Med.

[26] Hu H, Krasinskas A, Willis J (2011). Perspectives on current tumor-node-metastasis (TNM) staging of cancers of the colon and rectum. Semin Oncol.

